# Association of depression with sexual function in women with history of recurrent pregnancy Loss: descriptive-correlational study in Tehran, Iran

**DOI:** 10.1186/s40738-020-00089-w

**Published:** 2020-12-08

**Authors:** Seyed Ali Azin, Fahimeh Golbabaei, J. Catja Warmelink, Sadaf Eghtedari, Shima Haghani, Fahimeh Ranjbar

**Affiliations:** 1 Reproductive Biotechnology Research Center, Avicenna Research Institute, ACECR, Tehran, Iran; 2 Nursing Care Research Centre, School of Nursing and Midwifery, Iran University of Medical Sciences, Rashid Yasemi St., Valiasr Ave, Tehran, Iran; 3 Department of Midwifery Science, Amsterdam Public Health Research Institute, VU University Medical Center, Amsterdam, The Netherlands; 4 Department of General Practice & Elderly Medicine, University Medical Centre Groningen, University of Groningen, Amsterdam, The Netherlands; 5 AVAG (Amsterdam/Groningen Midwifery Academy), Amsterdam, The Netherlands

**Keywords:** Depression, Recurrent pregnancy loss, Recurrent miscarriage

## Abstract

**Purpose:**

The present study aimed to investigate the relationship between depression and sexual function in women with recurrent pregnancy loss.

**Methods:**

In a cross-sectional correlational study, 130 consecutive patients with history of recurrent pregnancy loss were included who referred to Avicenna Fertility Center in Tehran, Iran during November 2018–February 2019. The outcomes were sexual dysfunction (Assessed with the Female Sexual Function Index) and depression (Evaluated with the Beck’s Depression Inventory). The study data were analyzed by using Mann-Whitney and Kruskal-Wallis tests.

**Results:**

The study findings revealed that 40.8% of the participants suffered from some degrees of depression. The data analysis revealed that depression had a significant inverse correlation with sexual function and its domains (r = − 0.392, *p* < 0.001, *R*^2^= 0.15). The spouse’ education level and economic status demonstrated a significant relationship with women’s sexual function (*p* = 0.01, *p* = 0.033). A significant relationship was also detected between women’s depression and economic status (*p* = 0.028).

**Conclusions:**

The study findings showed that women with RPL who had severe depression indicated lower score of sexual function. Since psychological and sexual problems are not reported to health care providers due to giving priority to fertility issues or considering such issues as taboos, the assessment of sexual and mental health needs to be part of the consultation in women with history of RPL, whether the patient seeks help for depression and sexual dysfunction or not.

## Introduction

The experience of pregnancy loss may alter women’s physical, psychological and mental health [1]. Pregnancy loss is associated with distress and anxiety which can have a significant emotional impact on women and their spouses [2], specifically within women who have experienced recurrent pregnancy loss (RPL) [3]. Depression, emotional stress, anxiety, fear and sleep disorders have been reported within women with RPL history [4–6]. In some cases, the psychological symptoms of anxiety and depression can persist up to one year [7]. In addition, the psychological consequences of previous miscarriage sustain in subsequent pregnancies [8]. Some studies have investigated the psychological consequences of RPL and revealed that depression is prevalent in women with a history of RPL [6, 9, 10]. The depression prevalence has been reported 41.3% within pregnant women with RPL history in a study carried out in Brazil [9]. A comparative study was also conducted in Iran and revealed that women who have undergone RPL, experience a lower quality of life as well as more depression and anxiety compared to women without a history of miscarriage [1].

It seems sexual dysfunction has a two-way relationship with depression [11]. Depression-related symptoms such as inability to enjoy, fatigue, and reduced self-esteem can impair one’s sexual function, and on the other hand, sexual dysfunction can indirectly result in infertility by decreasing the number of sexual intercourses [12, 13]. A study by Fabre and Smith showed that with increasing severity of depression, sexual dysfunction becomes more severe in women [14]. Results of a study conducted in Australia suggests that presence of any risk factors such as anxiety, depression and sexual dysfunction increases the likelihood of one or two other disorders in the future [15]. Depression is also strongly associated with decreased libido, dyspareunia, and orgasmic disorder [16, 17]. Even in the absence of clinical evidence of depression, negative mood can lead to sexual dysfunction [18, 19] and positive or negative sexual experience can affect one’s mood throughout the day [16].

Some studies have proposed contradictory results regarding sexual function and depression in women with a history of RPL which indicates the need for further research. A study in Portugal revealed that pregnant women with an RPL history reported more sexual dysfunction than the control group and greater severity of depression led to increased sexual dysfunction, as well [20]. In contrast, the results of another study carried out in Portugal indicated that couples’ relationships after miscarriage were similar to those before miscarriage and couples had less problems with respect to talking about sexual issues after miscarriage [21].

The prevalence of RPL in Iran is unclear but it seems that women suffering from RPL or infertility face with more psychological problems and lower quality of life compared to fertile women [22, 23] because Middle Eastern societies such as Iran are family oriented, with a high value placed on childbearing [2]. Furthermore, the prevalence of major depressive disorders is 4.1% in Iran and women are 1.95 times more likely to have major depressive disorder [24]. Iran is also in the early stages of treating sexual problems and facing a large number of patients who have low sexual knowledge [25]. Since talking about sexual matters is seen as a taboo and discussion about sexual issues is avoided, sexual counseling is often not provided to Iranian women [26, 27]. As sexuality plays an important role in people’s life as well as their wellbeing and family relationships, more researches in the field of sexual health is needed in Iranian setting. Few studies have been carried out on sexual function within women with a history of RPL and to the researchers’ knowledge, no study has been conducted in Iran with respect to investigating the association between sexual function and depression in women with history of RPL. For this reason, in this study, an attempt was made to explore the association between depression and female sexual function in the context of recurrent pregnancy loss.

## Materials and methods

The present study was a descriptive-correlational study. Participants were recruited through convenient sampling. Participants were 130 married women with a history of RPL, who referred to Avicenna Fertility Center in Tehran, Iran during November 2018–February 2019. This center is a semi-public center operating under the supervision of the Academic Center for Education, Culture, and Research (ACECR) and providing specialized fertility care in Iran.

In the current study, RPL was defined as the spontaneous loss of two or more clinical pregnancies consecutively before 24 weeks of gestation [2]. Married, non-pregnant Iranian women aged of 21–45 who had two or more consecutive pregnancy losses before 24 weeks of gestation with the minimum interval of six months since the last miscarriage were included in the study. Women who had undergone infertility treatment cycles (IO, IUI, IVF, ICSI) in the past three months or had history of diseases affecting the sexual function or depression were not included. Also, women who initiated their infertility treatment cycles were excluded because these women were potentially at higher risk of sexual dysfunction [28–30].

### Instruments

Participants were requested to fill the demographic/reproductive characteristic checklist, Female Sexual Function Index (FSFI), and Beck’s Depression Inventory (BDI) after an explanation and giving informed consent.

Demographic/reproductive checklist included independent variables such as age, spouse’s age, education level, spouse’s education level, job status, spouse’s occupation, economic situation, duration of marriage, contraception method, history of infertility, type of RPL, and the time of the last miscarriage.

FSFI was used to assess the participants’ sexual function in the previous 4 weeks. The FSFI has 19 items which was first introduced by Rosen (2000) to assess six domains of sexual desire (1 and 2), sexual arousal (3–6), vaginal lubrication (7–10), orgasm (11–13), satisfaction (14–16), and pain (17–19) [31]. Likert scale for each subscale ranging from 0 or 1 to 5 was used in which higher scores indicate better sexual function. The total FSFI score (2 to 36) was obtained by summing the scores of the six domains. Higher scores indicate a better sexual functioning with a 28 or lower cut off score indicative of impaired sexual function according to a study in Iran [32]. The validity and reliability of the Persian version of the tool was confirmed by Fakhri et al. [33]. The sexual dysfunction’s prevalence in participants was reported in another paper in detail (Under review).

The Beck’s Depression Inventory consists of 21 questions based on Diagnostic and Statistical Manual of Mental Disorders, 5th Edition (DSM-5) criteria in order to assess emotional, cognitive, motivational and physiological aspects of depression. Using a four-point Likert scale ranging from zero to three points, the maximum score was 63 and the minimum score 0. A score of less than 10, 10–18, 19–29, and 29–63, respectively demonstrate minimal depression, mild depression, moderate depression and severe depression [34]. This questionnaire was translated into Persian by Ghassemzadeh et al., according to which, internal consistency of 87% and test-retest reliability of 73% were obtained [35]. This questionnaire has also been used in Iran for women with a history of RPL [23].

### Sample size

G-Power software was used for calculating the sample size at 95% confidence interval [36] with power of 80%; the minimum sample size of 85 was needed to achieve correlation coefficient of at least 0.3. Accordingly, a total of 130 participants were included in current study.

### Data analysis

Statistical analysis was performed using SPSS software, version 16 and *P* < 0.05 was considered statistically significant. Continuous and categorical variables were displayed as means ± standard deviation (SD) and percentages, respectively. The Kolmogorov-Smirnov test was used to check the normality of quantitative variables and Mann-Whitney and Kruskal–Wallis tests were used to investigate the relationship between FSFI and BDI with demographic variables. The Spearman correlation coefficient was used to investigate the relationship between FSFI and BDI.

### Ethical approval

Ethical approval was achieved from the Ethics Committee of Iran University of Medical Sciences (IR.IUMS.REC 1396.31743). Women were asked to give written informed consent for their participation. Participants were informed that they are free to participate and can withdraw from the study at any time and this would not influence the quality of care received in this center. Questionnaires were completed anonymously observing the essentials of secrecy and confidentiality of patients and only identifiers were used for each questionnaire.

## Results

In total, 130 women who met the inclusion criteria participated in the study. The mean age of the participants and their husbands was 31.53 ± 5.11 and 35/74 ± 5/13 years, respectively. In this study, the majority of the participants were housewives (73.1%), and more than half of them had university education (55.4%). In addition, 72.3% of participants had moderate economic status and 50.8% of them had marriage duration of 5–9 years. A total of 70.8% of participants had primary RPL, 39.2% had history of infertility and 50.8% were using contraception. Also, the time of the last miscarriage was < 12 months in 50.8% of participants. Socio-demographic and reproductive characteristics of the participants are presented in detail in Table 1.

**Table 1:** The Relationship between participants’ Characteristics with Female Sexual Function index and Depression

Demographic Characteristics	Categories	Number (percentage)	FSFI		Depression	
			Median(95% confidence interval)	Test result	Median(95% confidence interval)	Test result
Education level	High school or lower	17 (13.1)	24.7 (23.4,29.1)	^††^*P* = 0.098	17 (12,27)	^††^*P* = 0.093
	Diploma	41 (32.3)	28.85 (26.8,29.7)		11 (6,14)	
	Higher education	71 (54.6)	27.9 (26.9,29.45)		11 (7,13)	
Job status	Housewife	95 (73.1)	27.9 (26.2,29)	^†^*P* = 0.669	12 (9,14)	^†^*P* = 0.569
	Employed	35 (26.9)	28.9 (26.4,29.8)		9 (5,18)	
Spouse’s education level	High school or lower	22 (17)	25.7 (23.4,29)	^††^P = 0.01	17 (10.5,23)	^††^*P* = 0.075
	Diploma	36 (27.7)	26.9 (25.5,28.4)		13 (9.5,14)	
	Higher education	72 (55.4)	29.2 (27.9,29.65)		8.5 (5,11.5)	
Spouse’s occupation	Self-employment	85 (65.4)	28 (26.15,29)	^†^*P* = 0.332	12 (9,14)	^†^*P* = 0.439
	Government job	45 (34.6)	28.6 (27.1,29.79)		11 (6,13)	
Economic situation	Undesirable	20 (15.4)	29 (25.5,29.45)	^††^P = 0.033	14 (10,22)	^††^P = 0.028
	Moderate	94 (72.3)	27.3 (26,28.85)		11.5 (9,13.5)	
	Desirable	16 (12.3)	30 (27.8,31.1)		4.5 (3,12.5)	
Duration of marriage (year)	5>	24 (18.5)	27.2 (25.3,29.6)	^††^*P* = 0.466	13.5 (7,18)	^††^*P* = 0.347
	5-9 yr	66 (50.8)	28.45 (26.1,29.19)		13 (9,14)	
	10-14 yr	40 (30.8)	28.6 (26.7,29.7)		7 (4,12)	
Contraception	No	64 (49.2)	28.9 (27.4,29.45)	^†^P = 0.46	11.5 (9,14.48)	^†^*P* = 0.704
	Yes	66 (50.8)	27.3 (25.9,28.95)		11 (7,13.5)	
History of infertility	No	79 (60.8)	27.9 (26.4,29.15)	^†^*P* = 0.683	11 (7,13)	^†^*P* = 0.094
	Yes	51 (39.2)	28.1 (26,29.2)		14 (9,17)	
Type of miscarriage	Primary	92 (70.8)	27.95 (26.2,29)	^†^*P* = 0.444	12.5 (9,15)	^†^*P* = 0.125
	Secondary	38 (29.2)	28.7 (26.4,29.8)		7 (5,13)	
Time of Last miscarriage (month)	12>	66 (50.8)	28.8 (27.2,29.2)	^††^*P* = 0.561	12 (9,14.5)	^††^*P* = 0.352
	12–24	33 (25.4)	28 (25.7,29.89)		9 (6,13)	
	24≤	32 (23.8)	27.1 (23.4,29.1)		13 (4,18)	
Age (years(	30>	48 (36.9)	26.5 (25.6,28.1)	^††^*P* = 0.18	11 (7,16)	^††^*P* = 0.976
	30–39	75 (57.7)	29 (27.6,29.6)		12 (8,13)	
	40≤	7 (5.4)	29.7 (16.55,31.55)		9 (4,32.5)	
Spouse’s age (years(	30>	13 (10)	26.6 (25.2,29.05)	^††^*P* = 0.782	11 (8.5,17)	^††^*P* = 0.315
	30–39	89 (68.5)	28.4 (26.4,29.1)		12 (9,14)	
	40≤	28 (21.5)	28.55 (24.71,29.75)		7.5 (2,13)	

†Mann-Whitney Test ††Kruskal-Wallis Test

The study findings revealed no relationship between sexual function and participants’ characteristics, except for economic situation (Med = 29.2, *P* = 0.01) and spouse’s education level (Med = 30.0, *P* = 0.033). Sexual function levels in women whose spouses had university education was significantly higher compared to women whose spouses’ education level was high school (*P* = 0.013) and diploma (*P* = 0.018). Moreover, sexual function level turned to be significantly higher than moderate within women with desirable economic status (Med = 14.0, *p* = 0.009) and this difference was not significant at other levels. It should be noted that no statistically significant relationship was detected between BDI and demographic/reproductive characteristics, except for economic level (*P* = 0.028). The economic status was the only variable that revealed a statistically significant relationship with depression; depression mean score obtained in women with undesirable economic status was significantly higher than that of women with desirable economic status (*P* = 0.007). There was no significant relationship between age of participants and age of their spouses with BDI (*P* = 0.686, *P* = 0.12). There was no significant relationship between age of participants and age of their spouses with FSFI (*P* = 0.297, *P* = 0.723) (Table 1).

**Fig. 1:**
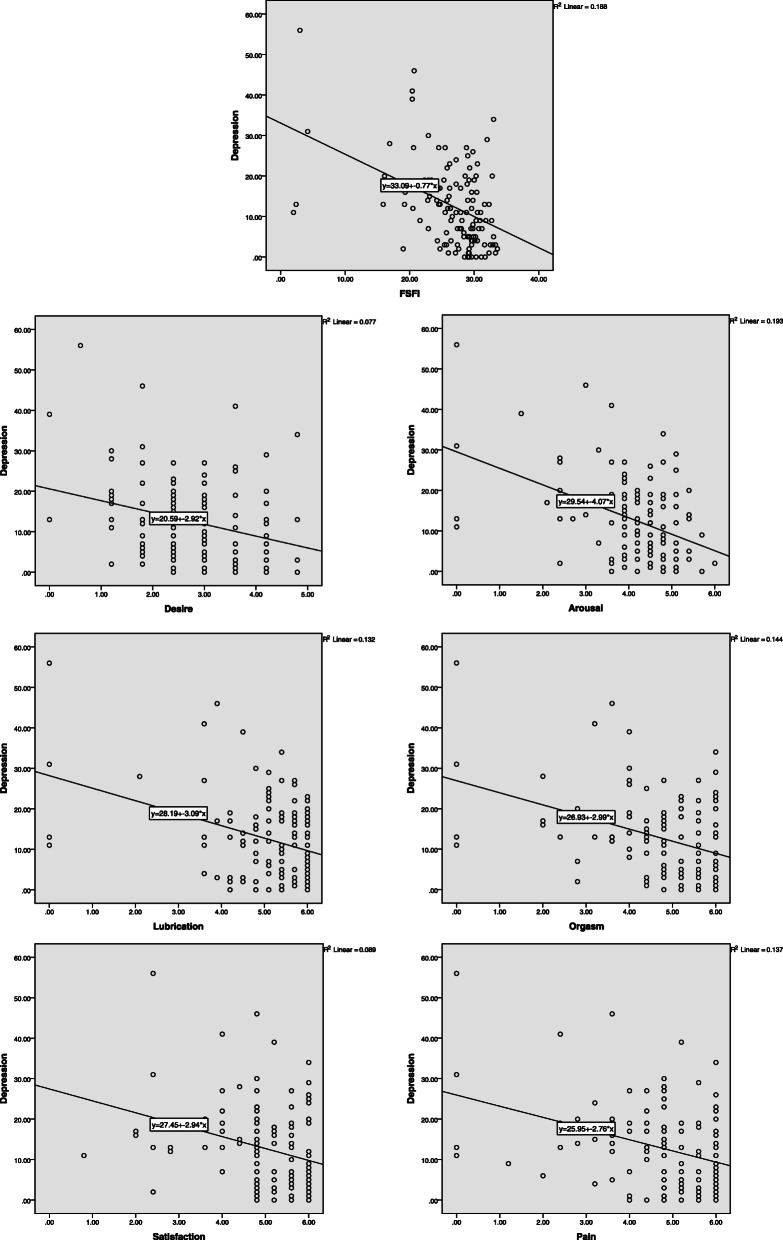
The scatterplot of FSFI score and its domain with BDI score in women with history of RPL

The study findings revealed that in total, the mean score of FSFI and BDI was 26.59 ± 5.73 and 12.61 ± 61.17, respectively. The results showed that 50.0% (*n* = 65) of the participants had sexual dysfunction. Rate of minimal, mild, moderate and severe depression were 59.2% (77), 21.5% (28), 13.1% (17) and 6.2% (8), respectively. Depression showed a statistically significant inverse correlation with FSFI and all of its domains; therefore, decreased scores of FSFI and all of its domains resulted in an increase in the BDI scores (*P* < 0.001, r = − 0.392, *R*^2^= 0.15). Given an r of − 0.392 between FSFI and depression (r^2^ = 0.15), 15% of the variance in depression may be attributed to differences among them in FSFI (Table 2). The inverse relationship between FSFI and its domains with BDI is outlined in Fig. 1.

**Table 2:** Mean and standard deviation of FSFI and BDI, and Spearman rho correlation between dimensions of FSFI and BDI

FSFI and its domains	Mean ± S.d	BDI
Desire	2.73 ± 0.96	r = −0.241 *P* = 0.006
Arousal	4.15 ± 1.09	r = −0.36 P < 0.001
Vaginal lubrication	5.04 ± 1.19	r = −0.22 *P* = 0.012
Orgasms	4.78 ± 1.29	r = −0.296 *P* = 0.001
Satisfaction	5.04 ± 1.03	r = −0.324 P < 0.001
Pain	4.83 ± 1.36	r = − 0.33 P < 0.001
FSFI (total)	26.59 ± 5.73	r = −0.392 *P* < 0.001
BDI	12.61 ± 10.17	–

*FSFI* Female sexual function index, *BDI* Beck Depression Inventory

## Discussion

In Iran, women react the same as other women in the world and the current study confirmed the high prevalence of sexual dysfunction and depression in women with history of RPL. The data analysis also revealed that FSFI and its domains had a statistically significant inverse correlation with BDI.

More than 40% of participants in the current study had mild to severe depression. Similarly, it has been reported that depression and emotional stress are extremely prevalent among women with RPL [5, 6]. The findings of a study conducted in Iran indicated that women with an RPL history has suffered from significant depression compared to the control group [1]. The findings of two studies conducted in Brazil also showed that more that 40% of women with an RPL history experienced depression [20, 37]. It seems that pregnancy loss has a direct relationship with psychological outcomes in at least some women [38] and a significant percentage of women suffering from RPL have reported a tendency for mental health counseling [39]. Therefore, depression screening should be a necessary part of every counseling visit for women with history of RPL. Health care providers should help Iranian women who avoid treatment due to social stigma associated with mental disease.

The findings showed a significant inverse relationship between BDI and FSFI as well as all its domains in women with history of RPL. Similarly, a study by Fabre and Smith showed that with increasing severity of depression, sexual dysfunction becomes more severe in women [14]. It seems that psychological factors play an important role with respect to sexual issues [9], and sexual dysfunction has a two-way relationship with depression. As a matter of fact, depression is associated with a 50–70% increase in sexual dysfunction, and sexual dysfunction increases the risk of depression by 130–200% [40]. Results of a study conducted in Australia suggests that presence of any risk factors such as anxiety, depression and sexual dysfunction increases the likelihood of one or two other disorders in the future [15]. Depression is also strongly associated with decreased libido, dyspareunia, and orgasmic disorder [16, 17]. Even in the absence of clinical evidence of depression, negative mood can lead to sexual dysfunction [18, 19] and positive or negative sexual experience can affect one’s mood throughout the day [16].

A study carried out in Portugal indicated that women with an RPL history suffered from depression twice as much as the control group and experienced severe sexual dysfunction; that is to say an increase in their depression score led to a decrease in their sexual function score [20]. Furthermore, in another study in Sweden, sexual dysfunction in women after termination of pregnancy was demonstrated to be associated with depression and anxiety [41]. The relationship between depression and sexual dysfunction in infertile women [22] and women suffering from polycystic ovary syndrome [42] has also been reported. It seems that childbearing difficulties and treatment-related stress can lead to problems in couples’ relationships as well as sexual dissatisfaction in this group of women [43]. Mental and sexual health have received scant attention in recurrent miscarriage because the health care providers focus only on the couple’s fertility. Sexuality has been surrounded by cultural restrictions, prohibitions, taboos, and indirect regulations in Iran [44]. The results of Javadnoori et al.’s study showed that taboo surrounding sexuality resulted in sexual silence, censorship, or emphasis on negative aspects of sexuality [45]. Therefore, since both depression and sexual dysfunction are common taboos in Iran, both issues can affect the quality of life; as a result, the assessment of sexual and mental health needs to be part of the consultation in women with history of RPL, whether the patient seeks help for depression and sexual dysfunction or not.

No relationship was detected between demographic/reproductive characteristics and sexual function, except for the economic situation and education level of spouse. Similarly, in a study in Iran that explored the relationship of economic and demographic factors with sexual and marital satisfaction in a sample of Iranian women, men’s educational level and finance index were correlated with their marital satisfaction. Linear regression showed that finance index had the strongest relationship with the sexual and marital satisfaction [46]. It seems that economic stressors of treatment negatively affect couples’ emotional and sexual intimacy. Highly-educated individuals also appear to have more appropriate occupational status, and fewer chronic stress who enjoy a healthier lifestyle and social support, and have better coping mechanisms and more skills, resulting in higher mental health [47]. Therefore, despite patriarchy in Iran, educated men can be more supportive of their spouses in the face of the socio-cultural pressures of infertility in family. Contrary to our findings, in a study conducted among Iranian infertile women, higher age of women, low education, unwanted marriage, short infertility duration, and low frequency of intercourse were related with sexual dysfunction [48].

The study findings illustrated depression had a statistically significant relationship with economic situation, that is to say women with undesirable economic situation exhibit more depression. Similarly, in a study by He et al., the low household income was a potential factor affecting depression and anxiety in patients with RPL [6]. Furthermore, it has been proposed that women with lower socio-economic status and those with a history of infertility or prior miscarriages are at greater risk of psychiatric morbidity following miscarriage [49].

In this study, all infertile patients were not excluded and 39.2% of the participants in the current study had infertility history. Hence, the high cost of infertility treatments in the absence of insurance coverage for infertility treatment in Iran may cause concerns in women with undesirable economic situation. Due to high value of childbearing in Iran, these women may feel depressed to a great extent about being childless than those who have a desirable economic situation. Therefore, women with poor economic status are more susceptible to depression; therefore, it seems necessary to provide more educational programs to teach them how to improve their mental health.

No relationship was found between termination of pregnancy and depression. Similar to our findings, Akker suggested that there is no evidence indicating a link between pregnancy termination and psychological reactions [50]. Keskin et al. also found that women with secondary infertility had decreased sexual function compared to women with primary infertility [51] but there wasn’t any relationship between primary or secondary RPL and sexual function in the current study [45].

Our study is the first study conducted in Iran and Middle East region with respect to investigating the association between sexual function and depression in women with history of RPL. One of the strengths of the current study was evaluation of patients from all over Iran who referred to recurrent miscarriage clinic in Tehran. However, the generalizability of the results seems to be difficult due to choosing only one referral miscarriage clinic. Considering limitations, no control group was included, which partially reduces the generalizability of our findings. This study suffers from another limitation which is lack of data about men. Moreover, further interventional research should be done to improve the mental and sexual health of the patients with a history of RPL.

## Conclusion

There is a high prevalence of sexual dysfunction and depression within women with a history of RPL. Our study suggests a significant inverse relationship between depression and sexual function within women with history of RPL. Since psychological and sexual problems are not reported to health care providers or therapists due to giving priority to fertility issues or considering such issues as taboos in Iran, it is considerably essential for health care providers to take into consideration the sexual and psychological matters in RPL patients, who refer to fertility or prenatal clinics, specifically women with low socio-economic status. The findings also confirm the necessity of training health care providers who are expert in the field of reproductive psychology and sexual health in management of patients with fertility problems in Iran.

## Data Availability

The datasets used and analyzed during the present study are available from the corresponding author on reasonable request.
